# Effect of weight change and lifestyle modifications on the development or remission of nonalcoholic fatty liver disease: sex-specific analysis

**DOI:** 10.1038/s41598-019-57369-9

**Published:** 2020-01-16

**Authors:** Naoki Yoshioka, Masatoshi Ishigami, Yasuko Watanabe, Hajime Sumi, Masao Doisaki, Takeo Yamaguchi, Takanori Ito, Yoji Ishizu, Teiji Kuzuya, Takashi Honda, Tetsuya Ishikawa, Jun-ichi Haruta, Mitsuhiro Fujishiro

**Affiliations:** 1 Department of Gastroenterology and Hepatology, Nagoya University Graduate School of Medicine, 65 Tsuruma-cho, Showa-ku Nagoya, 466-8550 Japan; 2 Department of Gastroenterology and Hepatology, Japanese Red Cross Nagoya Daiichi Hospital, 3-35 Michishita-cho, Nakamura-ku Nagoya, 453-8511 Japan; 3 Health control Center, Japanese Red Cross Nagoya Daiichi Hospital, 3-35 Michishita-cho, Nakamura-ku Nagoya, 453-8511 Japan

**Keywords:** Non-alcoholic fatty liver disease, Non-alcoholic steatohepatitis

## Abstract

The effects of changes in various lifestyle habits on nonalcoholic fatty liver disease (NAFLD) have not been well elucidated. We aimed to clarify how weight change and lifestyle modifications were associated with the development or remission of NAFLD. In this longitudinal cohort study, we reviewed the periodic health checkup data of 1,421 subjects with no causes of liver disease besides NAFLD who had received at least two health checkups between 2009 and 2018. The prevalence of NAFLD at baseline was 34.1% (484/1,421). During follow-up period (4.6 ± 2.8 years), 104 subjects developed NAFLD and 127 subjects demonstrated NAFLD remission. The frequency of NAFLD development or that of NAFLD remission significantly increased as the larger weight gain or weight loss was, respectively (both, p < 0.001). Approximately 40% of the subjects who maintained ≥ 1%/year weight loss achieved NAFLD remission. By multivariate analysis, quitting smoking were independently associated with NAFLD development (adjusted odds ratio [AOR], 2.86; 95% CI, 1.24–6.62). Subjects who quit smoking demonstrated large weight gain (≥1%/year) significantly more frequently than the other subjects (p < 0.001). In sex-specific analysis, starting to exercise was independently associated with NAFLD remission in men (AOR, 2.38; 95% CI, 1.25–4.53).

## Introduction

Nonalcoholic fatty liver disease (NAFLD) is one of the most common chronic liver diseases and will become the leading cause of end-stage liver disease in the near future. NAFLD is a global public health concern with a heavy healthcare burden^1^.

NAFLD encompasses a spectrum of progressive pathological conditions, ranging from nonalcoholic fatty liver (NAFL) to steatohepatitis (NASH)^2^. Many clinical trials have shown that weight loss and lifestyle intervention improve markers of liver function and histological features in patients with NAFLD^3–13^. Thus, NAFLD is closely related to lifestyle habits, and is often described as the hepatic manifestation of metabolic syndrome.

NAFLD is reversible to some extent. There have been several studies on lifestyle habits associated with the development or remission of NAFLD^14–18^. However, most of the studies focused only on the lifestyle habits at baseline, but not the changes in lifestyle habits during the study period.

Beginning in April 2008, Japan initiated a change in the national health policy in order to prevent metabolic syndrome. This comprehensive preventive policy involves unprecedented nationwide screening and lifestyle intervention for abdominal obesity, and the effectiveness of the nationwide preventive program has been demonstrated^19^. In addition, various lifestyle habits can be modified as a result of recently growing health consciousness across Japanese society.

Understanding the effects of various lifestyle modifications on NAFLD is important for educating the general public as well as for providing guidance to practitioners regarding health checkups. In the present study, we investigated how weight change and lifestyle modifications were associated with the development or remission of NAFLD.

## Results

### Study population

**Figure 1:**
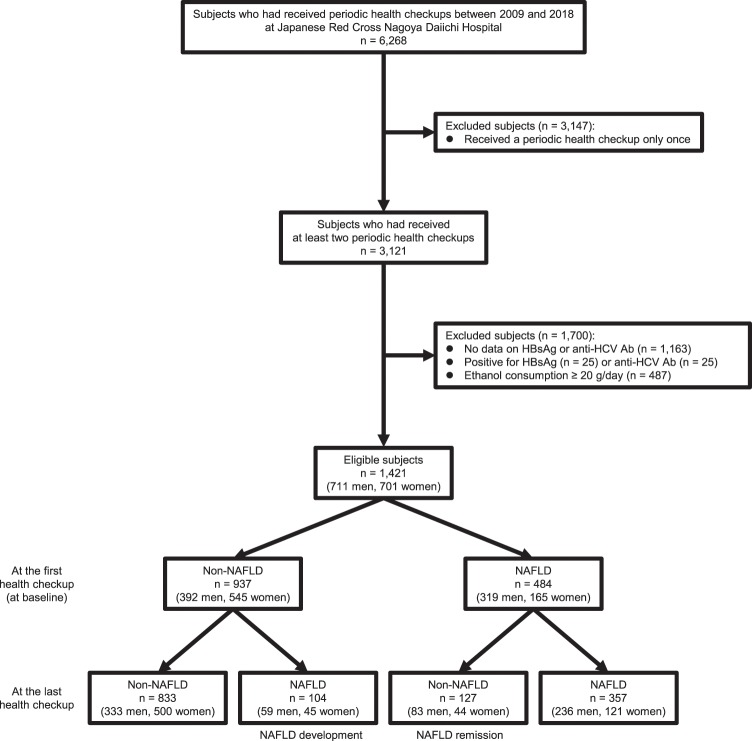
Flow chart of the study population. Eligible subjects comprised 1,421 individuals with no causes of liver disease other than nonalcoholic fatty liver disease (NAFLD) who had received at least two health checkups between 2009 and 2018. Among them, 104 subjects developed NAFLD and 127 subjects demonstrated NAFLD remission during the follow-up period. HBsAg, hepatitis B surface antigen; anti-HCV Ab, anti-hepatitis C virus antibody.

In this longitudinal cohort study, we reviewed the health checkup records of 6,268 Japanese adults who had received periodic health checkups between January 2009 and December 2018 at Japanese Red Cross Nagoya Daiichi Hospital (Fig. 1). Among them, 3,121 subjects had received at least two health checkups during this time. Then, subjects who were positive for hepatitis B surface antigen (HBsAg) or anti-hepatitis C virus antibody (anti-HCV Ab) and those who had not undergone these tests were excluded. Further, subjects who drank ≥ 20 g/day of ethanol were excluded. Finally, a total of 1,421 subjects (711 men and 710 women) was eligible for the study.

### Characteristics of subjects at baseline

At the first health checkup (at baseline), 484 of 1,421 (34.1%) subjects were diagnosed with NAFLD (Fig. 1). In the subjects with NAFLD at baseline, aspartate transaminase (AST), alanine aminotransferase (ALT), and platelet levels, AST to platelet ratio index (APRI), and body mass index (BMI) were significantly higher than those without NAFLD (Table 1). The frequencies of male gender, fatty liver index (FLI) ≥ 30, hypertension, dyslipidemia, diabetes mellitus, smoking, and eating dinner within 2 hours of going to bed were significantly higher and that of exercise was significantly lower in subjects with NAFLD than those without NAFLD.

**Table 1:** Characteristics of subjects at baseline.

	All (n = 1,421)	Non-NAFLD (n = 937)	NAFLD (n = 484)	p value Non-NAFLD *vs*. NAFLD
Age (years)	53.0 ± 11.9	52.8 ± 12.6	53.3 ± 10.5	0.476
Male gender (yes)	711 (50.0)	392 (41.8)	319 (65.9)	<0.001
AST (U/L)	22.8 ± 8.6	21.3 ± 6.7	25.8 ± 10.8	<0.001
ALT (U/L)	23.0 ± 14.9	18.2 ± 8.4	32.3 ± 19.6	<0.001
Platelet (×10^9^/L)	237 ± 65	231 ± 69	249 ± 56	<0.001
APRI	0.27 ± 0.13	0.26 ± 0.11	0.29 ± 0.15	<0.001
CRP (mg/dL)	0.10 (0.00–0.10)	0.03 (0.00–0.11)	0.10 (0.04–0.16)	0.470
FLI ≥ 30 (yes)	432 (30.4)	114 (12.2)	318 (65.7)	<0.001
FIB-4 index ≥ 2.67 (yes)	26 (1.8)	24 (2.6)	2 (0.4)	0.003
BMI (kg/mm^2^)	22.7 ± 3.5	21.4 ± 2.7	25.3 ± 3.4	<0.001
Hypertension (yes)	252 (17.7)	128 (13.7)	124 (25.6)	<0.001
Dyslipidemia (yes)	196 (13.8)	107 (11.4)	89 (18.4)	<0.001
Diabetes mellitus (yes)	55 (3.9)	29 (3.1)	26 (5.4)	0.042
Exercise (yes)	328 (23.1)	236 (25.2)	92 (19.0)	0.010
Smoking (yes)	222 (15.6)	123 (13.1)	99 (20.5)	<0.001
Eating dinner within 2 hours of going to bed (yes)	305 (21.5)	175 (18.7)	130 (26.9)	<0.001

Continuous variables except for CRP are expressed as means ± standard deviations. CRP is expresssed as medians (interquartile ranges). They were compared by the unpaired t test. Categorical variables are expressed as numbers (percentages), and were compared by the chi-squared test. ALT, alanine aminotransferase; APRI, aspartate aminotransferase to platelet ratio index; AST, aspartate aminotransferase; BMI, body mass index; CRP, C-reactive protein; FIB-4 index, fibrosis-4 index; FLI, fatty liver index; NAFLD, nonalcoholic fatty liver disease.

### Comparison of characteristics of subjects between baseline and the last health checkup

During the follow-up period (4.6 ± 2.8 years), the number of health checkups which subjects had received was 4.8 ± 2.7. During the period, 104 of 937 (11.1%, 2.4%/year) subjects without NAFLD at baseline developed NAFLD (Fig. 1). In contrast, 127 of 484 (26.2%, 5.7%/year) subjects with NAFLD at baseline demonstrated NAFLD remission. Therefore, as shown in Table 2, the prevalence of NAFLD decreased from 34.1% at baseline to 32.4% at the last health checkup, although the difference was not statistically significant (p = 0.148). ALT levels decreased significantly (p = 0.034). The frequency of exercise increased significantly, while that of smoking decreased significantly (both, p < 0.001).

**Table 2:** Comparison of characteristics of subjects between baseline and the last health checkup.

	At baseline (n = 1,421)	At the last health checkup (n = 1,421)	p value
Age (years)	53.0 ± 11.9	57.6 ± 12.4	<0.001
NAFLD (yes)	484 (34.1)	461 (32.4)	0.148
AST (U/L)	22.8 ± 8.6	23.0 ± 9.1	0.425
ALT (U/L)	23.0 ± 14.9	22.3 ± 14.1	0.034
Platelet (×10^9^/L)	237 ± 65	231 ± 59	<0.001
APRI	0.27 ± 0.13	0.28 ± 0.15	0.004
CRP (mg/dL)	0.10 (0.00–0.10)	0.06 (0.02–0.10)	0.187
FLI ≥ 30 (yes)	432 (30.4)	437 (30.8)	0.772
FIB-4 index ≥ 2.67 (yes)	26 (1.8)	60 (4.2)	<0.001
Body weight (kg)	60.7 ± 12.4	60.8 ± 12.4	0.876
Exercise (yes)	328 (23.1)	418 (29.4)	<0.001
Smoking (yes)	222 (15.6)	176 (12.4)	<0.001
Eating dinner within 2 hours of going to bed (yes)	305 (21.5)	288 (20.3)	0.284

A total of 1,421 subjects (711 men and 710 women) was eligible for the present study. Continuous variables except for CRP are expressed as means ± standard deviations. CRP is expresssed as medians (interquartile ranges). They were compared by the paired t test. Categorical variables are expressed as numbers (percentages), and were compared by the McNemar test. ALT, alanine aminotransferase; APRI, aspartate aminotransferase to platelet ratio index; AST, aspartate aminotransferase; CRP, C-reactive protein; FIB-4 index, fibrosis-4 index; FLI, fatty liver index; NAFLD, nonalcoholic fatty liver disease.

### Factors associated with NAFLD development

The changes in biochemical parameters and lifestyle habits were compared between subjects who developed NAFLD and those who remained non-NAFLD among subjects without NAFLD at baseline. As shown in Table 3, in subjects who developed NAFLD, ALT levels and the frequency of FLI ≥ 30 at the last health checkup were significantly higher than those who remained non-NAFLD. Gender (p = 0.001), follow-up period (p = 0.017), AST change (p = 0.023), ALT change (p < 0.001), and the frequencies of dyslipidemia (p = 0.013), diabetes mellitus (p = 0.034), weight loss (p < 0.001), and quitting smoking (p = 0.013) differed significantly between those who developed NAFLD and those who remained non-NAFLD. Among the changes of biochemical parameters, FLI change was most significantly associated with NAFLD development (Supplementary Table S1).

**Table 3:** Characteristics of subjects who developed NAFLD or demonstrated remission.

	Non-NAFLD at baseline (n = 937)			NAFLD at baseline (n = 484)		
	Non-NAFLD at the last health checkup (n = 833)	NAFLD at the last health checkup (n = 104)	p value	Non-NAFLD at the last health checkup (n = 127)	NAFLD at the last health checkup (n = 357)	p value
Age (years)	52.9 ± 12.6	52.4 ± 12.6	0.721	55.0 ± 11.3	52.7 ± 10.2	0.037
Male gender	333 (40.0)	59 (56.7)	0.001	83 (65.4)	236 (66.1)	0.913
Follow-up period (years)	4.7 ± 2.8	5.4 ± 2.8	0.017	4.9 ± 2.7	4.1 ± 2.7	0.003
ALT at the last health checkup (U/L)	17.4 ± 6.7	24.4 ± 12.9	<0.001	21.3 ± 10.1	33.4 ± 20.6	<0.001
FLI ≥ 30 at the last health checkup (yes)	92 (11.0)	51 (49.0)	<0.001	54 (42.5)	240 (67.2)	<0.001
FIB-4 index ≥ 2.67 at the last health checkup (yes)	43 (5.2)	1 (1.0)	0.079	5 (3.9)	11 (3.1)	0.578
AST change (U/L)	−0.1 ± 6.3	1.5 ± 7.3	0.023	−2.0 ± 7.9	0.8 ± 13.9	0.038
ALT change (U/L)	−0.4 ± 7.5	3.1 ± 12.6	<0.001	−7.1 ± 15.4	−0.2 ± 18.7	<0.001
Platelet change (×10^9^/L)	−5.2 ± 51.8	−1.2 ± 30.6	0.433	−5.3 ± 36.5	−10.6 ± 38.7	0.177
APRI change	0.00 ± 0.09	0.02 ± 0.09	0.169	−0.02 ± 0.10	0.03 ± 0.20	0.011
CRP change (mg/dL)	−0.04 ± 0.72	0.02 ± 0.24	0.453	−0.04 ± 0.82	0.00 ± 0.34	0.411
Hypertension (yes)	109 (13.1)	19 (18.3)	0.171	33 (26.0)	91 (25.5)	0.906
Dyslipidemia (yes)	87 (10.4)	20 (19.2)	0.013	24 (18.9)	65 (18.2)	0.894
Diabetes mellitus (yes)	22 (2.6)	7 (6.7)	0.034	11 (8.7)	15 (4.2)	0.067
Weight loss (yes)	376 (45.2)	21 (20.2)	<0.001	89 (70.1)	159 (44.5)	<0.001
Started exercising (yes)	107 (12.8)	10 (9.6)	0.432	28 (22.0)	43 (12.0)	0.008
Quit smoking (yes)	27 (3.2)	9 (8.7)	0.013	6 (4.7)	14 (3.9)	0.795
Stopped eating dinner within 2 hours of going to bed (yes)	63 (7.6)	4 (3.8)	0.224	14 (11.0)	39 (10.9)	1.000

Univariate analysis was performed. Continuous variables are expressed as means ± standard deviations, and were compared by the unpaired t test. Categorical variables are expressed as numbers (percentages), and were compared by the chi-squared test. ALT, alanine aminotransferase; APRI, aspartate aminotransferase to platelet ratio index; AST, aspartate aminotransferase; CRP, C-reactive protein; FIB-4 index, fibrosis-4 index; FLI, fatty liver index; NAFLD, nonalcoholic fatty liver disease.

Multivariate analysis showed that male gender (adjusted odds ratio [AOR], 2.07; 95% confidence interval [CI], 1.34–3.21), longer follow-up period (AOR, 1.11; 95% CI, 1.03–1.20), presence of dyslipidemia (AOR, 2.39; 95% CI, 1.25–4.56), absence of weight loss (AOR, 0.30; 95% CI, 0.18–0.50), and presence of quitting smoking (AOR, 2.86; 95% CI, 1.24–6.62) were independently associated with the development of NAFLD (Table 4).

**Table 4:** Factors associated with NAFLD development or remission.

	Association of NAFLD development (n = 937)			Association of NAFLD remission (n = 484)		
	AOR	95% CI	p value	AOR	95% CI	p value
Age	0.99	0.97–1.00	0.130	1.02	1.00–1.04	0.085
Male gender	2.07	1.34–3.21	0.001	0.97	0.61–1.53	0.892
Follow-up period	1.11	1.03–1.20	0.006	1.12	1.03–1.21	0.005
Hypertension	1.27	0.66–2.47	0.477	0.76	0.43–1.32	0.331
Dyslipidemia	2.39	1.25–4.56	0.008	0.87	0.45–1.67	0.671
Diabetes mellitus	1.98	0.75–5.24	0.169	1.88	0.75–4.72	0.178
Weight loss	0.30	0.18–0.50	<0.001	2.83	1.80–4.44	<0.001
Started exercising	0.64	0.31–1.31	0.222	1.66	0.95–2.89	0.074
Quit smoking	2.86	1.24–6.62	0.014	1.04	0.37–2.95	0.941
Stopped eating dinner within 2 hours of going to bed	0.43	0.15–1.26	0.124	1.20	0.60–2.37	0.607

Multivariate analysis was performed using a logistic regression model, adjusted for all other factors in the table. AOR, adjusted odds ratio; NAFLD, nonalcoholic fatty liver disease.

**Figure 2:**
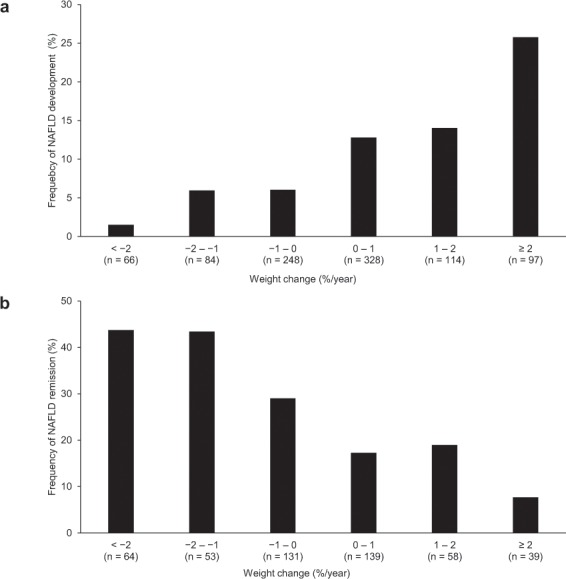
The association of weight change with NAFLD development and remission. (**a**) The larger weight gain was, the higher the frequency of NAFLD development was (p < 0.001). (**b**) The larger weight loss was, the higher the frequency of NAFLD remission was (p < 0.001).

**Figure 3:**
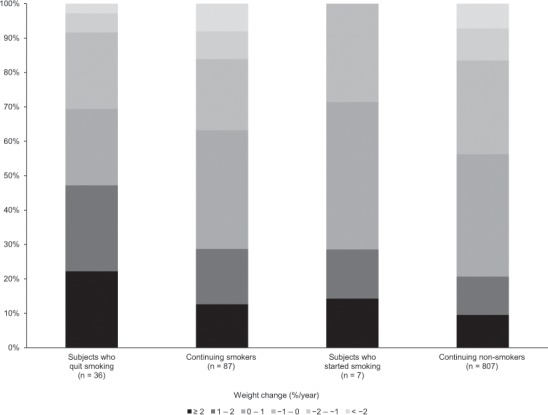
Weight change of subjects without nonalcoholic fatty liver disease at baseline, categorized according to changes of smoking habit. Subjects who quit smoking demonstrated large weight gain (≥1%/year) significantly more frequently than the other subjects (p < 0.001).

The association of weight change with NAFLD development was further analyzed. The larger weight gain was, the higher the frequency of NAFLD development was (p < 0.001) (Fig. 2a). As shown in Fig. 3, the subjects who quit smoking during the follow-up period demonstrated large weight gain (≥1%/year) significantly more frequently than the other subjects, including subjects who started smoking, continuing smoker, and non-smoker (p < 0.001).

### Factors associated with NAFLD remission

The changes in biochemical parameters and lifestyle habits were compared between subjects who demonstrated NAFLD remission and those remained NAFLD among subjects with NAFLD at baseline. As shown in Table 3, in subjects who demonstrated NAFLD remission, ALT levels and the frequency of FLI ≥ 30 at the last health checkup were significantly lower than those remained NAFLD. Age (p = 0.037), follow-up period (p = 0.003), AST change (p = 0.038), ALT change (p < 0.001), APRI change (p = 0.011), and the frequencies of weight loss (p < 0.001) and starting to exercise (p = 0.008) differed significantly between those who demonstrated NAFLD remission and those who remained NAFLD. Among the changes of biochemical parameters, FLI change (p < 0.001) and APRI change (p = 0.011) were independently associated with NAFLD remission (Supplementary Table S1).

Multivariate analysis showed that follow-up period (AOR, 1.12; 95% CI, 1.03–1.21) and weight loss (AOR, 2.83; 95% CI, 1.80–4.44) were independently associated with NAFLD remission (Table 4). There was a mild but nonsignificant association between NAFLD remission and both older age (AOR, 1.02; 95% CI, 1.00–1.04) and starting to exercise (AOR, 1.66; 95% CI, 0.95–2.89). In sex-specific analysis, only in men, the independent association between NAFLD remission and starting to exercise was present (Table 5).

**Table 5:** Factors associated with NAFLD development or remission in sex-specific analysis.

	men						women					
	Association of NAFLD development (n = 392)			Association of NAFLD remission (n = 319)			Association of NAFLD development (n = 545)			Association of NAFLD remission (n = 165)		
	AOR	95% CI	p value	AOR	95% CI	p value	AOR	95% CI	p value	AOR	95% CI	p value
Age	0.98	0.95–1.00	0.070	1.03	1.00–1.06	0.023	1.00	0.97–1.03	0.982	0.99	0.95–1.03	0.475
Follow-up period	1.20	1.08–1.34	<0.001	1.13	1.03–1.25	0.013	1.02	0.91–1.14	0.767	1.07	0.93–1.24	0.353
Hypertension	1.30	0.51–3.32	0.584	0.70	0.36–1.37	0.300	1.40	0.53–3.72	0.500	0.76	0.26–2.28	0.629
Dyslipidemia	2.64	1.00–6.97	0.050	1.11	0.51–2.44	0.788	1.71	0.65–4.49	0.279	0.48	0.13–1.73	0.263
Diabetes mellitus	1.02	0.24–4.31	0.978	1.78	0.60–5.29	0.301	4.51	1.03–19.80	0.046	1.36	0.20–9.28	0.753
Weight loss	0.13	0.05–0.32	<0.001	2.36	1.34–4.15	0.003	0.54	0.27–1.06	0.075	4.44	1.98–9.94	<0.001
Started exercising	0.47	0.17–1.33	0.154	2.38	1.25–4.53	0.009	0.87	0.32–2.38	0.788	0.41	0.12–1.49	0.177
Quit smoking	2.88	0.97–8.55	0.056	1.22	0.41–3.64	0.720	3.42	0.85–13.80	0.083	0.00	0.00–Infinity	0.989
Stopped eating dinner within 2 hours of going to bed	0.27	0.06–1.23	0.091	1.34	0.60–3.02	0.480	0.84	0.19–3.71	0.821	1.00	0.25–4.03	0.997

Multivariate analysis was performed using a logistic regression model, adjusted for all other factors in the table. AOR, adjusted odds ratio; NAFLD, nonalcoholic fatty liver disease.

The association of weight change with NAFLD remission was further analyzed. The larger weight loss was, the higher the frequency of NAFLD remission was (p < 0.001) (Fig. 2b).

## Discussion

The present study showed that the prevalence of NAFLD at baseline was associated with lifestyle habits, such as exercise, smoking, and eating dinner within 2 hours of going to bed, in addition to BMI, gender, hypertension, dyslipidemia, and diabetes mellitus. All these lifestyle habits associations were reported previously^14,15,20–23^. Other lifestyle habits, including sleep duration and sitting time, have also been reported to be associated with NAFLD^24,25^.

Many clinical trials have shown that patients with NAFLD benefit from weight loss^3–6^ and either dietary^7–9^ or exercise-related^10–13^ lifestyle interventions. There have been several longitudinal cohort studies on lifestyle habits involved in the development or remission of NAFLD. However, most of these studies assessed lifestyle habits at baseline but not changes in these habits during the study period^14,16–18,26^. We conducted a longitudinal cohort study to investigate the association of the development or remission of NAFLD with favorable changes in the lifestyle habits during follow-up period: weight loss, starting to exercise, quitting smoking, and stopping eating dinner within 2 hours of going to bed.

This study showed that change of body weight was significantly associated with both the development and remission of NAFLD. Quitting smoking was significantly associated with NAFLD development. In men, starting to exercise was significantly associated with NAFLD remission.

Several cohort studies have reported that change in body weight was significantly associated with both the development and remission of NAFLD^27,28^. The present study showed that weight change was correlated with both the frequency of NAFLD development and that of NAFLD remission. The frequency of NAFLD remission reached a plateau of 43% in subjects with 1–2%/year weight loss, and that of subjects with ≥ 2%/year weight loss was similarly 44%. Musso *et al*. reported that ≥ 7% weight loss also improved histological disease activity in NASH^4^. However, the most effective rate of weight loss has not been well elucidated. Wong *et al*. conducted a clinical trial in which patients with NAFLD were provided with a 12-month lifestyle intervention, and then followed up for the next 5 years without intervention^6^. In patients with a baseline BMI of ≥ 25 kg/m^2^, the mean change in body weight from baseline was −7.0 ± 4.8 kg at the end of the intervention, but only −1.9 ± 4.7 kg after 5 years. ALT levels temporarily dropped once but rebounded to the baseline level. This suggests that it is important to maintain the reduced body weight. Although the present study did not assess either the timing or speed of weight loss, it showed that approximately 40% of subjects who maintained ≥ 1% weight loss during the 4.6-year follow-up period achieved NAFLD remission.

In this study, quitting smoking was independently associated with NAFLD development. This finding can be attributed to the significantly higher frequency of weight gain in subjects who quit smoking compared with the other subjects. Hamabe *et al*. reported that the odds ratio of NAFLD development among new quitters was 2.73 compared with nonsmokers^14^. Considerable weight gain after quitting smoking^29^ is probably due to increased appetite and reduced energy expenditure^30^. Hu *et al*. showed that the risk of type 2 diabetes was higher among recent quitters than among current smokers, and that the increase in the risk of type 2 diabetes was directly proportional to weight gain^31^. The same mechanism is probably applicable to NAFLD development after quitting smoking. Quitting smoking decreases both cardiovascular and all-cause mortality^31^. Thus, the recommendation of quitting smoking is important, while the guidance is necessary not to gain body weight and to avoid NAFLD development.

Starting to exercise was mildly but nonsignificantly associated with NAFLD remission in the analysis among all subjects of the present study. However, in men, starting to exercise was independently associated with NAFLD remission. Osaka *et al*. also reported in a 10-year cohort study that regular exercise at baseline, defined as participating in any kind of sports at least once a week, was associated with NAFLD remission only in men. Several clinical trials have shown the effectiveness of exercise in patients with NAFLD^10,12^. Further studies are needed to elucidate the difference between genders in terms of the effect of exercise on NAFLD.

The present study showed that eating dinner within 2 hours of going to bed was significantly associated with the prevalence of NAFLD, while stopping of eating dinner within 2 hours of going to bed was not associated with either remission or development of NAFLD. Bo *et al*. showed that consuming a higher proportion of one’s daily energy intake at dinner was associated with an increased risk of NAFLD, obesity, and metabolic syndrome^26^. This suggests that eating habits affect NAFLD development. More detailed data on eating habits are needed in order to investigate the association between eating habits and NAFLD.

The prevalence of NAFLD at baseline in the present study was 34.1% (484/1,421), which is higher than that of published studies in Japan (9–30%)^32,33^. The subjects who were positive for HBsAg or anti-HCV Ab, or who drank ≥ 20 g/day of ethanol were excluded from the present study. When these subjects were included to the mother population, the prevalence of NAFLD is 24.7% (484/1,958). Among the subjects who had received health checkup only once during our study period and were excluded from the study, the prevalence of NAFLD was 30.9% (971/3,147). Thus, the prevalence of NAFLD of our study population was similar to that of general population or that of the population who were not eligible in the present study.

Our study population might have a relatively high health awareness, as indicated by the fact that the proportion of subjects with favorable lifestyle habits such as non-smoking and exercise significantly increased during the follow-up period, which allowed us to investigate the association of various lifestyle modifications with the development and remission of NAFLD. Moreover, we took advantage of the availability of the relatively large cohort and long follow-up period.

Our study had several limitations. First, since data on lifestyle habits were based on a self-reported questionnaire, it was uncertain when lifestyle habits were changed and how long these habits were continued. Second, the diagnosis of NAFLD was based on ultrasonography. Ultrasonography is a widely accepted imaging technique for the detection of fatty liver, because the both sensitivity and specificity of ultrasound for the detection of moderate-severe fatty liver compared to histology were high (84.8% and 93.6%, respectively)^34^. However, it may not detect mild steatosis and cannot differentiate NAFL from NASH. When diagnosing of fatty liver by ultrasonography, the four ultrasonographic findings (parenchymal brightness, liver-to-kidney contrast, deep beam attenuation, and vascular blurring) have been used. In the present study, we adopted the guideline for the ultrasonographic diagnosis of fatty liver published by the Japan Society of Ultrasonics in Medicine, in which fatty liver is diagnosed when one or more of the four findings^35^. However, the specificity of this protocol may be lower especially in obese subjects, because subjects with thick layers of subcutaneous fat can have deep beam attenuation, despite the absence of hepatic steatosis. The combination of the four findings can enable higher specificity. We used FLI, APRI, and fibrosis-4 (FIB-4) index to assess hepatic steatosis and fibrosis. FLI was significantly associated with the prevalence of NAFLD diagnosed by ultrasonography and its change was most significantly associated with the development and remission of NAFLD diagnosed by ultrasonography among the changes of biochemical parameters. This may indicate that the diagnostic method of NAFLD by ultrasonography is reliable. The value of APRI was low in most of the subjects. The subjects with FIB-4 index ≥ 2.67 are suspected to have advanced fibrosis and therefore NASH^36^. Among 484 subjects with NAFLD at baseline, the number of subjects with FIB-4 index ≥ 2.67 was only two (0.4%) at baseline and 16 (3.3%) at the last checkup. Among the 16 subjects, two (one remained NAFLD and one demonstrated NAFLD remission) had FIB-4 index ≥ 2.67 at baseline and 14 (10 remained NAFLD and 4 demonstrated NAFLD remission) grew worse to have FIB-4 index ≥ 2.67 at the last checkup. Among 104 subjects with NAFLD development, none had FIB-4 index ≥ 2.67 at baseline, and one grew worse to have FIB-4 index ≥ 2.67 at the last checkup. These results suggested that the number of subjects with NASH was very small at baseline, but increased at the last checkup in the present study. It is needed to assess other fibrosis markers, liver stiffness or biopsy for the study of NASH in the future. Third, the ultrasonographic grade of hepatic steatosis was not considered in the present study. Mustapic *et al*. reported that patients with higher ultrasonographic grade of hepatic steatosis significantly more frequently had metabolic syndrome, while the grade of hepatic steatosis did not correlate with the presence of hepatic fibrosis^37^. However, there have been no reports on the clinical importance of the reduction in the number of ultrasonographic findings of NAFLD. It was difficult to define the boundary condition in the reduction in the number of ultrasonographic findings since the baseline numbers differed among the subjects. Thus, we set the boundary between none and one or more of the four characteristic ultrasonographic findings in NAFLD. Several cohort studies have also showed that disappearance of ultrasonographic findings for NAFLD was associated with a decreased risk of developing subclinical atherosclerosis and type 2 diabetes^38,39^. The clinical importance of the reduction in the number of ultrasonographic findings should be elucidated in the future studies.

In conclusion, the present longitudinal cohort study showed that weight change was correlated with both the frequency of NAFLD development and that of NAFLD remission. Quitting smoking induced NAFLD development, probably due to weight gain. Finally, starting to exercise was independently associated with NAFLD remission in men.

## Methods

### Study assessments

The health checkup included a standard questionnaire, physical examination, biochemical tests, and abdominal ultrasonography. The questionnaire assessed each patient’s medical history of hypertension, dyslipidemia, and diabetes mellitus, as well as lifestyle habits such as alcohol consumption (how often and how much do you drink?), exercise (have you regularly exercised for over 30 minutes at a time, until you sweat lightly, at least twice weekly for over a year?), smoking (have you smoked during the past 6 months?), and eating dinner within 2 hours of going to bed (do you eat dinner within 2 hours before bedtime 3 or more times a week?). Height and weight were measured without shoes or heavy clothing, and BMI (kg/m^2^) was calculated. APRI and FIB‐4 index were calculated by the following formula, respectively: APRI = (AST/upper limit of normal × 100)/platelet, FIB‐4 index = (age × AST)/(platelet × ALT^1/2^). The upper limit of normal AST levels was 38 U/L. FLI was calculated using BMI (in kg/m^2^), waist circumference (WC, in cm), triglycerides (TG, in mg/dL) and gamma-glutamyl transferase (GGT, in U/L): FLI = [e^(0.953 × ln (TG) + 0.139 × BMI + 0.718 × ln (GGT) + 0.053 × WC − 15.745)^]/[1 + e^(0.953 × ln (TG) + 0.139 × BMI + 0.718 × ln (GGT) + 0.053 × WC − 15.745)^] × 100, where ln is the natural logarithm and e Euler’s number.

NAFLD was defined as fatty liver without excessive alcohol consumption (ethanol consumption ≥ 20 g/day) or other chronic liver disease such as hepatitis B or C. In accordance with guideline published by the Japan Society of Ultrasonics in Medicine^35^, the diagnosis of fatty liver was required one or more of the following four ultrasonographic findings: parenchymal brightness, liver-to-kidney contrast, deep beam attenuation, and vascular blurring. Medical technologists performed all ultrasonography procedures using the Aplio SSA-700A (Toshiba, Tokyo, Japan) or Xario 200 Platinum (Canon, Tokyo, Japan). Two gastroenterologists who were blinded to the subjects’ clinical data reviewed all the ultrasonographic images and made the diagnosis of fatty liver (positive for one or more of the four characteristic ultrasonographic findings) or non-fatty liver (positive for none of the four findings). When two opinions varied, the diagnosis was decided by their discussion (kappa value for the intra-observer reliability was 0.980; 95% CI, 0.972–0.988). NAFLD development was defined as the transition from non-NAFLD (positive for none of the four characteristic ultrasonographic findings) to NAFLD (positive for one or more of the four findings) during the follow-up period which was defined as the period between the first and last health checkups. NAFLD remission was defined as the transition from NAFLD (positive for one or more of the four findings) to non-NAFLD (positive for none of the four findings) during the period.

We examined the factors associated with development or remission of NAFLD by comparing each patient’s data at the first health checkup (at baseline) with those at the last health checkup. With respect to changes in lifestyle habits at the last health checkup from baseline, “started exercising”, “quit smoking”, “stopped eating dinner within 2 hours of going to bed”, and “lost weight” were defined based on changes during the follow-up period. The average of weight change (%/year) was calculated as follows: (weight change during the follow-up period/body weight at baseline)/the follow-up period. Additionally, sex-specific analysis was performed on the factors associated with development or remission of NAFLD.

The study protocol including the method of opt-out consent was approved by the Institutional Review Board at Nagoya University Graduate School of Medicine (2019–0024) and Japanese Red Cross Nagoya Daiichi Hospital (2018–238), and the study was conducted in accordance with the concepts outlined in the Declaration of Helsinki. Informed consent was obtained in the form of opt-out on the website of Japanese Red Cross Nagoya Daiichi Hospital.

### Health guidance

The results of health checkup were given to all subjects on the checkup day at Japanese Red Cross Nagoya Daiichi Hospital. In addition, health guidance was provided to all subjects unless they denied it, by healthcare staff, consisting of physician, public health nurse, or nutritionist. Independent advice was given using leaflets. When received the final results by mail within 2 weeks after health checkup, specific health guidance was recommended for subjects in high risk with metabolic syndrome. Subjects who requested were provided additional continuous support for 3 months or longer in response to individual risks via interview or telephone.

### Statistical analysis

Continuous variables except for CRP were expressed as means ± standard deviations. CRP was expressed as medians and interquartile ranges. They were analyzed using the unpaired and paired *t* tests. Categorical variables were expressed as numbers and percentages, and were analyzed using the chi-squared test and the McNemar test. The Cochran-Armitage test was used to assess the trend in the frequencies of development or remission of NAFLD based on weight change. Multivariate analysis was performed using a logistic regression model. After adjusting for age, gender, follow-up period, hypertension, dyslipidemia, diabetes mellitus, starting exercise, quitting smoking, stopping eating dinner within 2 hours of going to bed, and weight loss, the AOR and 95% CI were calculated. The data were analyzed using EZR (Saitama Medical Center, Jichi Medical University, Saitama, Japan), which is a graphical user interface for R (The R Foundation for Statistical Computing, Vienna, Austria, version 3.5.3). More precisely, it is a modified version of the R commander (version 2.5–1) designed to add statistical functions frequently used in biostatistics^40^. All the reported p values were two-tailed, and a value of p < 0.05 was considered statistically significant.

## Supplementary information

Supplementary Table S1.
