# Supplementary material for: Effect of weight change and lifestyle modifications on the development or remission of nonalcoholic fatty liver disease: sex-specific analysis
Source: Sci Rep. 2020 Jan 16;10:481. doi: 10.1038/s41598-019-57369-9 (PMC6965633; doi:10.1038/s41598-019-57369-9)
Supplement: Supplementary file 1 — Supplementary Table S1. [file 41598_2019_57369_MOESM1_ESM.pdf]

Supplementary information

Effect of weight change and lifestyle modifications on the development or remission of nonalcoholic fatty liver disease: sex-specific analysis

Authors:

Naoki Yoshioka [1,2], Masatoshi Ishigami [1]*, Yasuko Watanabe [3], Hajime Sumi [2], Masao Doisaki [2], Takeo Yamaguchi [2], Takanori Ito [1], Yoji Ishizu [1], Teiji Kuzuya [1], Takashi Honda [1], Tetsuya Ishikawa [1], Jun-ichi Haruta [2], and Mitsuhiro Fujishiro [1]

Affiliations:

[1] Department of Gastroenterology and Hepatology, Nagoya University Graduate School of Medicine, 65 Tsuruma-cho, Showa-ku, Nagoya, 466-8550, Japan. Tel: +81-52-744-2169; [2] Department of Gastroenterology and Hepatology, Japanese Red Cross Nagoya Daiichi Hospital, 3-35, Michishita-cho, Nakamura-ku, Nagoya, 453-0046, Japan. Tel: +81-52-481-5111; [3] Health control Center, Japanese Red Cross Nagoya Daiichi Hospital, 3-35, Michishita-cho, Nakamura-ku, Nagoya, 453-0046, Japan. Tel: +81-52-481-5111

***Corresponding author:**

Masatoshi Ishigami, Nagoya University Graduate School of Medicine, 65 Tsuruma-cho, Showa-ku, Nagoya, 466-8550, Japan. Tel: +81-52-744-2169; Fax: +81-52-744-2178. E-mail: masaishi@med.nagoya-u.ac.jp

Supplementary Table S1. Biochemical parameters associated with NAFLD development or remission.

	Association of NAFLD development (n = 937)			Association of NAFLD remission (n = 484)		
	AOR	95% CI	p value	AOR	95% CI	p value
AST change	0.99	0.90–1.09	0.829	1.12	1.01–1.23	0.025
ALT change	1.06	1.01–1.10	0.019	0.99	0.97–1.02	0.540
Platelet change	1.00	0.99–1.00	0.578	1.00	0.99–1.01	0.538
APRI change	0.29	0.00–87.40	0.674	0.00	0.00–0.15	0.011
CRP change	1.33	0.79–2.23	0.284	0.75	0.48–1.15	0.181
FLI change	1.05	1.03–1.08	<0.001	0.96	0.94–0.98	<0.001

Multivariate analysis was performed using a logistic regression model, adjusted for all other factors in the table. ALT, alanine aminotransferase; AOR, adjusted odds ratio; APRI, aspartate aminotransferase to platelet ratio index; AST, aspartate aminotransferase; CRP, C-reactive protein; FLI, fatty liver index; NAFLD, nonalcoholic fatty liver disease.
